# A Genome-Wide Association Study and Genomic Prediction for Fiber and Sucrose Contents in a Mapping Population of LCP 85-384 Sugarcane

**DOI:** 10.3390/plants12051041

**Published:** 2023-02-24

**Authors:** Haizheng Xiong, Yilin Chen, Yong-Bao Pan, Ainong Shi

**Affiliations:** 1 Department of Horticulture, University of Arkansas, Fayetteville, AR 72701, USA; 2 USDA-ARS, Sugarcane Research Unit, Houma, LA 70360, USA

**Keywords:** sugarcane (*Saccharum* spp.), fiber content, sucrose content, genome-wide association study, genomic prediction and selection

## Abstract

Sugarcane (*Saccharum* spp. hybrids) is an economically important crop for both sugar and biofuel industries. Fiber and sucrose contents are the two most critical quantitative traits in sugarcane breeding that require multiple-year and multiple-location evaluations. Marker-assisted selection (MAS) could significantly reduce the time and cost of developing new sugarcane varieties. The objectives of this study were to conduct a genome-wide association study (GWAS) to identify DNA markers associated with fiber and sucrose contents and to perform genomic prediction (GP) for the two traits. Fiber and sucrose data were collected from 237 self-pollinated progenies of LCP 85-384, the most popular Louisiana sugarcane cultivar from 1999 to 2007. The GWAS was performed using 1310 polymorphic DNA marker alleles with three models of TASSEL 5, single marker regression (SMR), general linear model (GLM) and mixed linear model (MLM), and the fixed and random model circulating probability unification (FarmCPU) of R package. The results showed that 13 and 9 markers were associated with fiber and sucrose contents, respectively. The GP was performed by cross-prediction with five models, ridge regression best linear unbiased prediction (rrBLUP), Bayesian ridge regression (BRR), Bayesian A (BA), Bayesian B (BB) and Bayesian least absolute shrinkage and selection operator (BL). The accuracy of GP varied from 55.8% to 58.9% for fiber content and 54.6% to 57.2% for sucrose content. Upon validation, these markers can be applied in MAS and genomic selection (GS) to select superior sugarcane with good fiber and high sucrose contents.

## 1. Introduction

Sugarcane is a commercially important crop in tropical and subtropical regions of the world, accounting for more than 80% of global sugar production [1]. In 2021, the global sugarcane plantation area was 27.49 million hectares, and sugarcane production reached 1988.3 million tons [2]. As a renewable resource, sugarcane has been the best energy crop, available as food (sugar, Jaggery, syrup), feed (green tops/leaves) and fertilizer (pressed mud) [3]. In recent years, researchers have analyzed the potential of residues from the processing industry (bagasse, leaves and shoot-tips) for the production of second-generation ethanol and cogeneration of electric power. The production of alternative energy sources and the establishment of the concept of bio-refining have also led to a rapid increase in the global demand for sugar cane [4]. To meet this growing demand, the development of new cultivars with high biomass and sugar yields is essential. Modern sugarcane varieties (*Saccharum* spp. hybrids) are an interspecific hybrid of *Saccharum officinarum* (2n = 80) and *S. spontaneum* (2n = 40–128) [5]. *S. officinarum* has a high sugar content, while *S. spontaneum* provides resistance to various diseases and abiotic stresses [6,7].

The process of improving sugar content in interspecific sugarcane hybrids through backcrossing, known as “Nobilization”, requires 3 to 6 generations of crosses to stabilize the genome and restore the high sugar trait [8]. This process initiated with a (2n + n) chromosome transmission mode and ends with =100–144 chromosomes [9]. Genome analysis via in situ hybridization shows that *S. officinarum* and *S. spontaneum* make up about 80% and 10–20% of the genome of modern cultivars, respectively, with 10% being recombinant chromosomes [7,9,10]. The total genome size of sugarcane hybrids is estimated to be about 10 Gb with 10 homologous linkage groups [11]. The complex polyploid and aneuploid feature of sugarcane hybrids presents challenges in genetic analysis and trait isolation, which can be addressed through the use of advanced statistical methods and genomic tools in breeding [12]. Current breeding efforts focus on improving sugar yield, disease and pest resistance, ratooning ability, cold tolerance and total biomass yield [3]. However, the development of a new sugarcane cultivar typically takes 12–15 years and involves annual evaluation of 60,000 to 250,000 seedlings [13,14]. The meiotic chromosome pairing in sugarcane is primarily bivalent, but within specific homologous groups, there is a complex and unbalanced pattern of mutual and systematic pairing [15]. Using molecular markers associated with relevant agronomic traits can aid in the selection of suitable parent plants and speed up the process of genetic improvement during breeding, ultimately reducing the time and cost of developing new varieties [16].

Linkage/family-based genetic mapping has been successful in identifying several quantitative trait loci (QTL) that have major or minor phenotypic effects on for both simple and complex traits [17]. However, linkage mapping has some limitations, including the difficulty in establishing a single population that simultaneously isolates multiple traits, the time-consuming process of establishing mapping populations, the lack of high-density mapping, and the overestimation of phenotypic variations by markers [15]. Despite the numerous reports on linkage analysis during the past three decades, few QTLs have been identified in sugarcane due to the lack of a clear allelic isolation pattern in sugarcane genomes, such as complete polysomy or complete disomy. Family-based linkage mapping mainly utilizes F_1_ populations, which can be limited in clarity due to the large number of genes, polyallelic and/or polygenic properties, underutilization of sugarcane gene pools and limited internal comparisons between genes [15,18]. These limitations can be overcome through marker-trait association (MTA) studies based on linkage disequilibrium (LD) using diverse genotypes that capture a wide range of allelic diversity, often referred to as association mapping (AM) [16,19]. Linkage disequilibrium mapping was initially used in human genetic studies as an alternative to marker-trait association identification in plants [20,21]. This approach has been found to be particularly useful in sugarcane due to the large number of LD present in the genome [22]. Researchers have used models corrected for population structure (Q) to identify markers for disease resistance and other traits [23]. For example, Wei et al. (2006) identified markers for resistance to smut, Pachymetra root rot, scalding and Fiji leaf gall using Q-modified models [24]. Similarly, using a Q-modified model, the significant MTA of sugarcane yield and sugar content were 43% and 38%, respectively [16]. Debibakas et al. (2014) identified six independent markers of sugarcane yellow leaf virus resistance using a mixed linear model (MLM) of Q-K (population structure—kin) combination [25]. Gouy et al. (2015) used the Q-K combination to evaluate the general linear model (GLM) and MLMs of MTA for 13 traits related to agricultural morphology, sucrose yield, bagasse content and disease resistance [23]. Banerjee et al. (2015) evaluated the role of MTA on various sucrose and yield traits [26]. In another study, Q-K analysis was used to identify four SSR markers associated with red rot resistance [27]. Recently, Ukoskit et al. (2019) used MLM on a diversity panel consisting of 200 germplasm accessions and identified two SSR markers associated with polarization (Pol) and sugar production [28]. SNP (Single Nucleotide Polymorphism) markers have been used and have shown great potential in sugarcane studies in sugarcane studies of genetic basis of various traits. The use of SNP markers has allowed researchers to identify specific DNA regions that are associated with these traits, providing insights into the genetic mechanisms underlying these important characteristics with higher genomic coverage [29]. However, sampling bias, technical requirements and the cost of SNP genotyping still represent barriers for some sugarcane researchers, particularly in low-resource settings [30,31,32].

Genomic prediction (GP) is a method of predicting the breeding value of an individual plant based on its genomic data [17,33]. GP has the potential to significantly expedite the breeding process by allowing breeders to select for traits of interest without the need for extensive phenotyping and field testing [34]. In a study by Olatoye and colleagues, the effectiveness of GP and MAS in predicting traits with different genetic structures and marker densities was evaluated. Both F_1_ and BC_1_ populations were used for lignocellulosic, biomass and disease resistance analyses. The results showed that GP had a higher prediction accuracy and the best performance in simulating character values. GP was able to identify more genotypes, with prediction accuracy for characters reaching 44–77% [35]. Deomano and colleagues conducted a selection experiment in sugarcane using three different commercial populations at different stages. They found that the genomic prediction model with marker data provided a higher prediction accuracy (25–45%) than the model with pedigree data alone [36]. Similarly, in a study involving 3984 individuals, Hayes et al. showed that GP had a prediction accuracy of 30–47% for cane yield, commercial cane sugar (%), fiber content and flowering traits [37]. Islam and colleagues used target enrichment sequencing to generate 8825 SNP markers from 432 sugarcane clones and found that the prediction accuracy of various GP models for brown and orange rust resistance ranged from 0.28 to 0.43 and 0.13 to 0.29, respectively. The inclusion of a known master gene for brown rust resistance as a fixed effect in the GP model also significantly reduced the minimum number of markers and the size of training populations [38].

A basic premise of genomics-assisted breeding is the identification of trait-associated molecular markers. A genetic linkage framework map containing AFLP, TRAP and SSR markers has been constructed using a self-progeny mapping population of LCP 85-384, the most popular sugarcane cultivar in Louisiana from 1997 to 2007 [39]. The objectives of this study were to perform genome-wide association to identify DNA markers associated with sucrose and fiber contents in the LCP 85-384 mapping population, and to assess the efficiency of genomic prediction in order to apply marker-assisted genomic selection in sugarcane breeding programs.

## 2. Results

### 2.1. Fiber and Sucrose Contents

**Figure 1:**
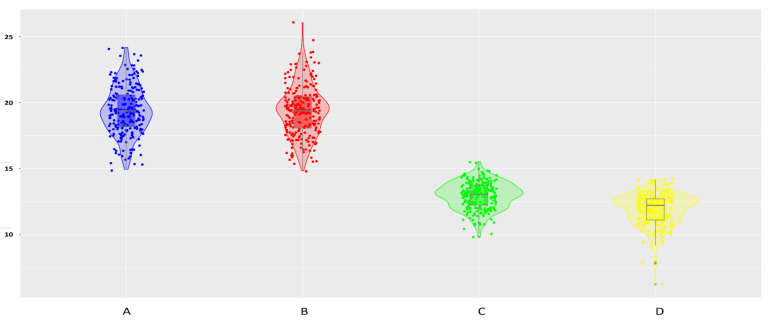
Combined violin boxplots of fiber and sucrose contents (%) over two years among 237 clones in the LCP 85-384 population: fiber contents in 2006 (blue) (**A**) and 2007 (red) (**B**), sucrose contents in 2006 (green) (**C**) and 2007 (yellow) (**D**).

The sucrose and fiber contents of the 237 clones were phenotyped for two years (Table S1). The statistical data and the distribution of the phenotypic traits are presented in Table 1 and Figure 1, respectively. The average of these values in 2006 was consistently higher than those in 2007, while the variance, standard error and coefficient of variation for the two traits were larger in 2007; however, these data were still at a low level in both years. The estimated broad-sense heritability values (*h*^2^) were high in both years (0.65~0.67 for sucrose and 0.74~0.77 for fiber). The same conclusion can be drawn from the population distribution of the two phenotypic data collected over two years in Figure 1. According to correlation analysis, sucrose and fiber contents were moderately negatively correlated (−0.23 and −0.40) for both years.

### 2.2. Genetic Relationship and Population Structure

**Figure 2:**
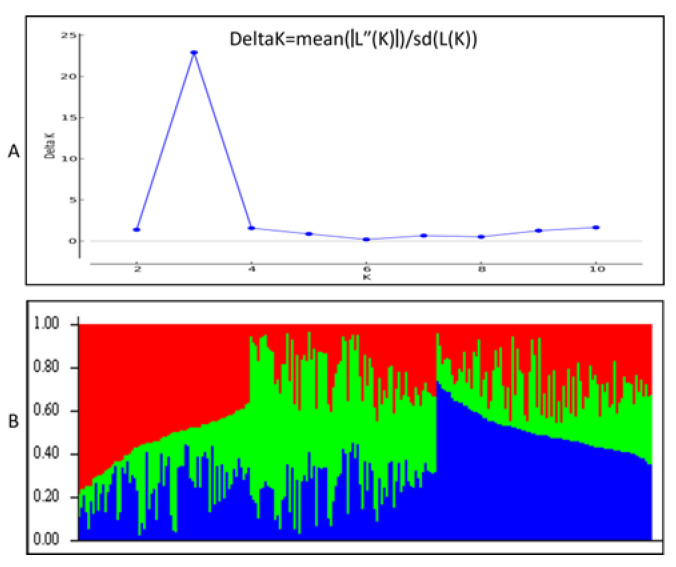
Structural analysis of 237 clones in the LCP 85-384 population based on 135 polymorphic markers (SSR, AFLP and TRAP). (**A**): Delta K values for different numbers of populations assumed (K = 10) in the STRUCTURE analysis. (**B**): Classification of 237 clones in three groups (K = 3) using STRUCTURE. The distribution of accessions to different sub-populations is color coded. The X-axis represent the 237 clones from the LCP 85-384 population and the value on the y-axis shows the likelihood of every individual belonging to one of the three colored subpopulations.

**Figure 3:**
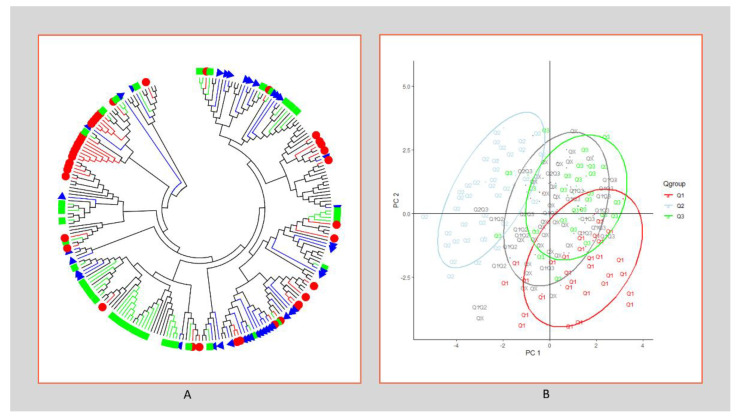
The phylogenetic and principal component analysis of 237 clones in the LCP 85-384 population based on 135 polymorphic markers (SSR, AFLP and TRAP). (**A**): Phylogenetic analysis of the 237 clones with the corresponding labels as Q groups according to the Structure results: Q1 = red pie, Q2 = green square, Q3 = blue triangle. (**B**): Scatter diagram of PCA for 237 clones in three color-coded Q groups (**A**) and mixed Q groups in gray color.

The population structure of the 237 self progenies was initially inferred using STRUCTURE 2.3.1 and the peak of delta K was observed at K = 3, by 135 polymorphic markers (Table S2) indicating the presence of three sub-populations (Figure 2A, Table S1). At a threshold value of 0.5, 41 of the 237 self progenies (17.2%) were assigned to the Q1 subpopulation; 47 self progenies (19.7%) were assigned to Q2; 39 self progenies (16.4%) assigned to Q3; Q1Q2 and Q2Q3 (Qx + Qy > 70%) were both assigned by 11 self progenies (4.6%); 26 self progenies (10.9%) were assigned to Q1Q3; and 62 self progenies (26.1%) were assigned to QX (Qx ≈ Qy ≈ Qz) (Figure 2B). Phylogenetic analysis and a population admixture map of the 237 self progenies using MEGA 11 software also showed a clustering pattern consistent with that inferred by structure K = 3 (Figure 3A). The most closely related self progenies based on Structure analysis were grouped in the neighbor branches of the phylogenetic tree by MEGA analysis. The three groups were also observed based on PCA dimensions (Figure 3B). Therefore, the 237 self progenies can be divided into three sub-populations based on both structural and phylogenetic analysis.

### 2.3. GWAS Analysis

**Figure 4:**
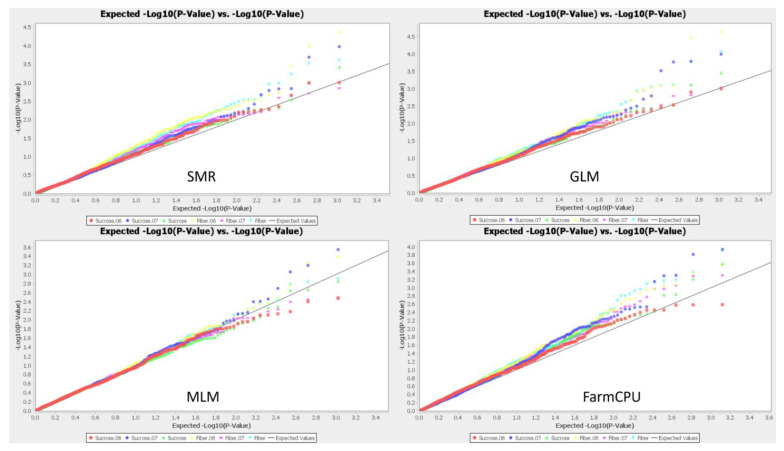
Graphs showing QQ-plots for sucrose and fiber contents collected in 2006 and 2007 by four GWAS models: SMR, GLM, MLM, and FarmCPU. Red Square: sucrose in 2006, Blue Pie: sucrose in 2007, Green Triangle: Mean sucrose 2006 and 2007; Yellow Diamond: fiber in 2006, Purple Rectangle: fiber in 2007, Cyan Inverted triangle: Mean fiber in 2006 and 2007.

GWAS analysis was conducted by using 1,310 polymorphic alleles (260 SSR, 950 AFLP and 100 TRAP) with four models: three models (SMR, GLM, MLM) in TASSEL 5 and FarmCPU in GAPIT 3. The QQ plots showed a large divergence from the expected distribution, indicating that there were alleles associated with both sucrose and fiber contents in the LCP 85-384 mapping population (Figure 4). In this study, 25, 18 and 25 alleles were significantly associated with fiber content (LOD > 2.0) of 2006, 2007 and the mean of 2006 and 2007, respectively; 13, 21 and 14 alleles were significantly associated with sucrose content of 2006, 2007 and the mean of 2006 and 2007, respectively, by using GLM analysis in TASSEL 5 (Table S3). Using MLM, 10, 11 and 11 alleles were associated significantly with fiber content of 2006, 2007 and mean of 2006 and 2007, respectively; and 7, 10 and 6 alleles were significantly associated with sucrose content of 2006, 2007 and the mean of 2006 and 2007, respectively (Table S3). As in SMR, 12, 15 and 12 markers were significantly associated with sucrose content of 2006, 2007 and mean of 2006 and 2007, respectively; 31, 19 and 23 markers were significantly associated with fiber content of 2006, 2007 and the mean of 2006 and 2007, respectively (Table S3). In addition, 8, 8 and 11 markers were significantly associated with sucrose content of 2006, 2007 and the mean of 2006 and 2007, respectively, and 5, 4 and 10 markers were significantly associated with fiber content of 2006, 2007 and mean of 2006 and 2007, respectively, using FarmCPU analysis (Table S3). After comprehensively measuring the LOD values and repeatability of the associated alleles across different models and years, 9 and 13 associated alleles were selected as association markers for sucrose and fiber contents, respectively (Table 2), which can be used in molecular breeding.

### 2.4. Genomic Prediction Analysis

**Figure 5:**
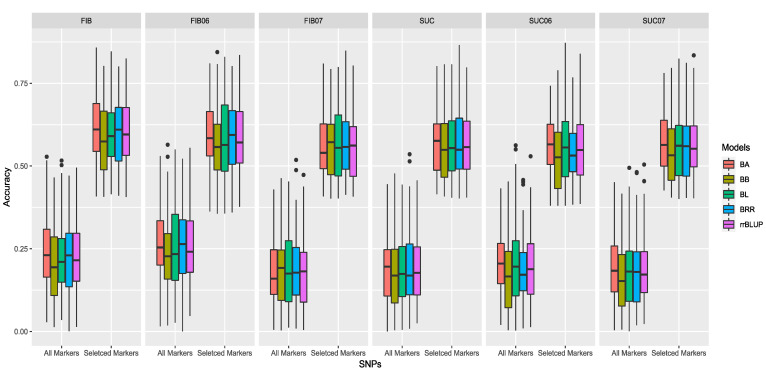
Genomic prediction (GP) accuracy (r-value) for fiber (FIB) and sucrose (SUC) using five GP models: Ridge regression best linear unbiased predictor (rrBLUP) = purple, Bayes ridge regression (BRR) = blue; Bayes A (BA) = red, Bayes B (BB) = dark yellow, and Bayesian least absolute shrinkage and selection operator (BL) = green, based on All-allele set and Trait-associated allele set.

In this study, two marker datasets, namely, the All-allele dataset and the Trait-associated-allele dataset, were used for GP analysis by five models: BA, BB, BL, BRR and rrBLUP (Figure 5). The predictive ability of each marker dataset was estimated using the sucrose and fiber content data in 2006, 2007 and the 2006 and 2007 mean. For sucrose contents, the All-allele dataset produced similar average accuracies among all models ranging from 16.4% (BB) to 20.1% (BA), as well as the average accuracies of fiber content ranging from 17.9% (BB) to 25.3% (BA). The average accuracies of the GWAS-associated allele set ranged from 55.8% (BB) to 58.9% (BA) for fiber contents and 54.6% (BB) to 57.2% (BA) for sucrose contents (Table S4). Compared with the All-allele dataset, the Trait-associated-allele set had higher accuracies in all models, 36.1% higher for fiber content and 37.4% higher for sucrose content, respectively. Therefore, using trait-associated marker alleles to perform GS is more efficient in selecting sucrose and fiber content in sugarcane breeding.

## 3. Discussion

### 3.1. Phenotyping

The cane stalks consist of a core containing most of the extractable sucrose, and the major component of outer layer is fiber containing lignocellulose. Improving sucrose or fiber content in cane stalks and balancing these two traits for practical industry have always been an important and challenging task for sugarcane breeders [40]. Increasing sucrose content while maintaining an acceptable level of fiber content in the stalk rather than increasing cane yield is seen as an economically viable option, as it can potentially avoid the increased costs associated with increased harvesting, transport and milling [1,41]. The heritability is crucial to predict the potential for improving those quantitative traits through selective breeding or other genetic interventions [42,43]. In this study, the broad-sense heritability estimates for the sucrose and fiber contents were high at 65~67% for sucrose and 74~77% for fiber, showing that genetic variation was a significant factor in the two traits, and indicating that it would be effective to improve the traits in selection. As expected, negative correlations (−0.23 and −0.40) were observed between sucrose and fiber contents during the two-year phenotyping experiments, indicating that the synthesis of sucrose in plants requires the breakdown of complex carbohydrates such as fiber [44].

### 3.2. The Self-Progeny Population of LCP 85-384

LCP 85-384 was a very popular cultivar in the Louisiana sugar industry due to its high sugar yield and various favorable agronomic traits, including tolerance to biotic and abiotic stresses. The sucrose and fiber contents of some self progenies were found to be higher than those of commercial sugarcane hybrids. The higher sucrose and fiber contents of LCP 85-384 may be attributed to the presence of *S. officinarum, S. spontaneum* and *S. barberi* clones in its pedigree [39]. LCP 85-384 has also been used extensively as a parent in Louisiana breeding programs. The genetic analysis of its self-progeny mapping population with AFLP, SSR and TRAP DNA markers has resulted in an enriched linkage map, and several alleles from these markers were found to be associated with sucrose or fiber content [45,46].

### 3.3. Genetic Diversity, Population Structure and PCA

Sugarcane is a complex polyploid plant species with a large genome size and a high level of genetic diversity [11]. The presence of sub-populations in a mapping population can create challenges for association studies, and methods such as STRUCTURE and PCA are often used to deal with false positives related to population structure [21]. These methods can be used to identify and control population structure in order to increase the power to detect true associations in a GWAS. It is also noted that the use of STRUCTURE may be limited in the case of sugarcane due to its complex polyploid genome and the lack of clear discontinuities in the population [16]. In this study, both PCA and STRUCTURE were used as a random component in the GWAS analysis to control for population structure and maximize the power to detect true associations. The results of the population structure analysis, the phylogenetic analysis and the distribution of genotypes on the PCA were all consistent with one another, suggesting a high level of consistency in the data.

### 3.4. Genome-Wide Association Study

The long breeding cycles in sugarcane have resulted in large blocks of linkage disequilibrium (LD), which can be advantageous for identifying markers associated with genes that regulate important agronomic traits [47]. However, the highly heterozygous genome of sugarcane and severe inbreeding depression for certain traits can make it difficult to study the F_1_ progeny of two heterozygous parents, or even the selfed progeny population of a selected cultivar [22]. That is why linkage mapping has limitations, such as inflating estimates and hard-to-find minor QTLs. Multiple models were developed, including SMR, GLM, MLM and FarmCPU, etc., for GWAS based on linkage disequilibrium. Nonetheless, the performance of these models can vary depending on the specific population, the trait being studied and the environment in which the study was conducted [48]. Previous research has shown that the differences in the performance of these models are often due to the interactions between the methods and other factors [49,50]. Each of these models has its own strengths and limitations, and researchers may comprehensively consider more models based on the specific research question and the data available. In this case, we applied four GWAS models to identify QTLs of fiber and sucrose contents with the LOD threshold > 2.0. These trait-associated QTLs (Table S3) were highly consistent with former reports, at 24/28 [45] in sucrose content and 2/3 in fiber contents [46]. Then, considering the consistency among models, 13 and 9 markers were selected as putative QTLs for fiber and sucrose contents, respectively. Of these, most of the alleles were AFLPs, while only two SSRs and one TRAP were associated with sucrose content and one SSR was associated with fiber. This is likely to be caused by differences in the number of markers and the level of polymorphism; therefore, the type of marker used can also affect the accuracy and reliability of GWAS results.

### 3.5. Genomic Prediction

Normally, genomic prediction (GP) does not work well for traits that are influenced by complex interactions between multiple genes and the environment. Therefore, the traits with high heritability, such as the sucrose and fiber contents in this study, would be more suitable for breeding value prediction by GP [51,52]. We selected the most popular and reliable GP models that have been applied to sugarcane, including one BLUP model and four Bayesian models in this case. The accuracies of GP based on a Trait-associated-allele set (Table S4) were higher than the All-allele set, which demonstrated the importance of significant alleles. It is worth noting that in all GP models, predictions for fiber content were higher than those for sucrose content, which is consistent with their heritability, demonstrating the reliability of the prediction.

The typical breeding cycle for sugarcane can vary depending on the specific cultivar, growing conditions and breeding method, but it can often last 12–15 years or even longer for a new cultivar, including time for seed production, growth, evaluation and selection [13,14]. In the last two years, researchers began to introduce the GP technology into sugarcane breeding, which involves many traits such as yield, bagasse, sucrose and disease resistance based on SNP markers [35,36,37,38]. For these applications, using DNA markers associated with specific traits of interest, breeders can identify individuals with desirable traits early in the growth cycle, before they become fully mature. This allows breeders to make their selections more quickly and helps reduce the time and labor consumption required to evaluate the crop. Additionally, GP and marker-assisted selection can help breeders to track progress more accurately while pursuing their breeding goals, as well as avoiding selections that are likely to negatively affect other traits. However, the use of large-scale sequencing or diversity array technology for molecular data analysis can be expensive and requires advanced technology. This study was the first to use SSR, AFLP and TRAP for GP analysis in sugarcane, which makes it more practical and reliable for breeding teams with limited resources [53,54].

## 4. Materials and Methods

### 4.1. Plant Materials

A mapping population of LCP 85-384 sugarcane with 237 genetically verified self progenies was used in this study. LCP 85-384 was developed from a cross between CP 77-310 and CP 77-407 through a collaboration among the Louisiana State University Ag Center, the USDA-ARS Sugarcane Research Unit, and the American Sugarcane League of the USA, Inc. [39].

### 4.2. Phenotyping and Analysis

Replicated field trials of the LCP 85-384 mapping population were conducted in 2006 (plant cane crop) and 2007 (first ratoon crop) at the Ardoyne Farm, USDA-ARS Sugarcane Research Unit, Schriever, Louisiana, USA (29°37′55.93″ N, 90°50′10.47″ W). In the field, each progeny was replicated and planted in a single 3 m-long plot, with 1.0 m between plots and 1.8 m between rows. Sucrose content and fiber data were measured by hand-cutting 10 millable stalks at ground level, removing the top just below the apical meristem, knife-stripping the stalks to remove leaf and sheath tissue, bundling and tagging the stalks and then weighing them to obtain an estimate of individual stalk weight (kg). The stalks were cut and shredded in a pre-breaker (Cameco Industries Inc., Thibodaux, LA, USA). Juice was extracted from a 1 kg shredded sample in a core press (Cameco Industries Inc., Thibodaux, LA, USA) under 211 kg/cm pressure in the juice quality laboratory to measure total soluble solids, Brix and sucrose content. The remaining “cake” of fibrous residues was weighed, then dried at 66 °C for 72 h to obtain the dry weight of the fibrous residues. The sucrose and fiber content was estimated according to the methods of Legendre and Henderson [55].

The phenotypic data were analyzed by SAS 9.2 (SAS 2008) software. The variance of fiber and sucrose contents was analyzed using the model of Aitken et al. (2006) [56]. Covariance analysis of sucrose and fiber contents was performed using various covariance components and mean product. The sources of variability between phenotypic datasets, as well as the system and error variances, as a proportion of the variance, were also calculated by dividing the system variance by the total variance for a particular trait. The formula of heritability (*h*^2^) was used to determine each trait.

(1) $$h^{2}=\frac{\sigma_{G}^{2}}{\sigma_{G}^{2}+\sigma_{\varepsilon }^{2}/r}$$

where $\sigma_{G}^{2}$ is the genetic variance, $\sigma_{\varepsilon }^{2}$ the residual variance and *r* the number of replicates.

### 4.3. Genetic Markers and Genotyping

The details of DNA extraction, genotyping and linkage map construction were previously described by Andru et al. (2011) [39] and Liu et al. (2016) [45]. In brief, genomic DNA was extracted from young leaves from each progeny by Pan et al. (2000) [57]. DNA concentrations were measured at 260 nm using a NanoDrop 1000 spectrophotometer (NanoDrop, Bethesda, MD, USA) and then equilibrated by agarose gel electrophoresis. Genotyping protocols including primer sequence, PCR amplification and PCR product detection for AFLP and TRAP markers were described in Andru et al. (2011) [39] and for SSR genotyping in Pan et al. (2007) [58]. The construction of an enriched genetic linkage map of LCP85-384 was described in Liu et al. (2016) [45]. In this study, we picked up the markers with less than 5% missing data and more than 2% minor frequency as genotyping data. In total, 60 SSR, 63 AFLP and 12 TRAP markers were used, including 260 polymorphic SSR alleles (4.3/marker), 950 polymorphic AFLP alleles (15/marker) and 100 TRAP polymorphic alleles (8.3/marker) (Table S2).

### 4.4. Genetic Diversity and Population Structure

All the polymorphic SSR, TRAP and AFLP alleles scored on the 237 self progenies were used to estimate their genetic relationship. STRUCTURE 2.3.1 was used to infer the population structure [59]. To identify the number of populations (K) and capture the major structure in the data, we set up at a burn-in period of 10,000 Markov Chain Monte Carlo iterations and 100,000 run length with an admixture model following Hardy–Weinberg equilibrium, correlated allele frequencies, as well as independent loci for each run. Ten independent runs were performed for each simulated K value, ranging from 1 to 10. For each simulated K, the statistical value delta K was calculated using the formula described by Evanno et al. [60]. The optimal K was determined using Structure Harvester [61]. Genetic dissimilarities between all pairwise combinations of progenies were calculated using the Dice index described by Shi et al. [62]. Then, a Neighbor Joining tree was built from the matrix of pairwise dissimilarities using the software MEGA 11 [63]. Phylogenetic relationships and principal component analysis (PCA) were generated by TASSEL 5.2.13 to analyze genetic relationships among progenies and to determine the optimal number of clusters in the study. The number of principal components (PC) was chosen according to the optimum subpopulation determined in STRUCTURE 2, and a PCA plot was drawn using R package ggplot2 using the data from TASSEL 5 [64]. The genetic diversity was also assessed, and phylogenetic trees were drawn using MEGA 11 based on the Maximum Likelihood tree method with the parameters described by Shi et al. [62]. During the drawing of the phylogeny trees, the population structure and the cluster information were imported for the combined analysis of genetic diversity.

### 4.5. Genome-Wide Association Study and Genomic Prediction

GWAS was performed for fiber and sucrose content in the self-progeny mapping population of LCP 85-384 using single marker regression (SMR), a generalized linear model (GLM), the mixed linear model (MLM) [65] by Tassel 5 and the fixed and random model circulating probability unification (FarmCPU) [66] of R software GAPIT 3 [67]. The significant threshold of associations was set when the LOD [−log(*p*-value)] > 2.0 in this study.

GP was conducted using two types of genotype datasets: all polymorphic alleles (1310) and traits associated alleles according to the GWAS results. All the alleles with LOD > 2.0 (Table S3) were considered and applied for prediction with the phenotype of the corresponding year. Genomic estimated breeding value (GEBV) was computed using 5 different statistical models, namely, Ridge regression best linear unbiased predictor (rrBLUP) [68], Bayes ridge regression (BRR), Bayes A (BA), Bayes B (BB), and Bayesian least absolute shrinkage and selection operator (BL) [69]. A five-fold cross validation to a training/testing set as 80%/20% was performed for the genomic prediction study. The association panel was randomly divided into five disjointed groups. A total of 100 replications were conducted at each fold. Mean and standard errors corresponding to each fold were computed [70].

## 5. Conclusions

Fiber and sucrose contents and genotyping data of the 237 self progenies of an LCP 85-384 mapping population were analyzed in this study. The results showed that the two traits have large genetic variances with a high broad-sense heritability: 68–69% for sucrose content and 82–85% for fiber content. GWAS identified 9 and 13 marker alleles as associated with sucrose and fiber contents, and GP estimated up to 57.2% and 58.9% prediction accuracy for sucrose and fiber content, respectively, when using a trait-associated-allele dataset. The study identifies GWAS-derived marker alleles significantly associated with sucrose and fiber contents and provides useful information for the breeders to use in marker-assisted and genomic selection programs.

## Figures and Tables

**Table 1:** Phenotypic data indicating mean, standard deviation (Std Dev), standard error (Std Err), min, max, range, variance, coefficient of variation (CV), median, broad-sense heritability (*h*^2^) and correlation coefficient (r) of sucrose and fiber contents in 2006 and 2007 among 237 clones in the LCP 85-384 population.

Traits	Year	Mean (%)	Std Dev	Std Err	Min (%)	Max (%)	Range (%)	Variance	CV (%)	Median (%)	Broad-Sense Heritability (*h*^2^)	Correlations (r)	
												Sucrose	Fiber
Sucrose	2006	12.98	1.02	0.07	9.84	15.54	5.70	1.04	7.87	13.07	0.65	1.00	−0.40
	2007	11.92	1.23	0.08	6.25	14.19	7.94	1.51	10.30	12.04	0.67	1.00	−0.23
Fiber	2006	19.42	1.89	0.13	14.96	24.17	9.21	3.58	9.75	19.27	0.74	−0.40	1.00
	2007	19.38	1.98	0.13	14.84	26.02	11.18	3.92	10.22	19.44	0.77	−0.23	1.00

**Table 2:** The molecular markers associated with sucrose and fiber contents in LCP 85-384 population based on four GWAS models: single marker regression (SMR), generalized linear model (GLM), mixed linear model (MLM) and fixed and random model circulating probability unification (FarmCPU) with data of 2006, 2007 and mean (2006 and 2007), where only LOD [−log(*p*-value)] > 2.0 were listed.

Marker	LOD [−log(*p*-Value)]												Trait
	SMR	GLM	MLM	FarmCPU	SMR	GLM	MLM	FarmCPU	SMR	GLM	MLM	FarmCPU	
	2006				2007				Mean (2006 and 2007)				
E32M49_110					3.31	3.30	3.05	3.05	3.66	3.59	3.38	3.44	Sucrose content
E32M50_290	2.33	2.59	2.42	2.44					2.89	2.84	2.46	2.71	
E32M61_246					2.56	2.41	2.26	2.36	2.23	2.20	2.05	2.09	
E32M62_76	2.23	2.46	2.04	2.14					3.29	3.21	2.50	3.10	
E37M49_183	2.29	2.58	2.77	2.20		2.30	2.20		2.45	2.82	2.92	2.30	
E39M50_82									2.03	2.12	2.04		
SMC703BS_214					2.35	2.48	2.27	2.17	2.07	2.09	2.03		
SMC703BS_216					2.35	2.48	2.27	2.17	2.07	2.09	2.03		
StSy-R3-128					2.17	2.21	2.13		2.23	2.23	2.18		
E32M49_431		2.45	2.22						2.36	2.68	2.40		Fiber Content
E32M61_127	3.67	2.71	2.11	2.70	2.60	2.08		2.25	3.84	3.09	2.19	3.11	
E33M61_97	2.98	2.94	2.33	2.20	2.95	2.97	2.63	2.55	3.32	3.37	2.59	2.70	
E33M62_170	2.66	2.44			2.32	2.17		2.00	3.32	3.16	2.19	2.70	
E36M48_61	4.18	3.55	2.89	3.08					3.28	2.95	2.40	2.66	
E36M60_250	2.33	2.11			2.26	2.13			2.95	2.84	2.62	2.39	
E36M61_73					3.26	3.28	3.54	2.81	2.10	2.13	2.27		
E37M50_316	2.89	3.07	2.19	2.13	2.79	2.77		2.41	3.81	3.90	2.72	3.09	
E38M61_165	3.77	3.36	2.62	2.78					3.37	3.19	2.19	2.73	
E40M59_188		2.25							2.08	2.37	2.06		
E40M62 153		2.19	2.40							2.07	2.17		
E41M61_215					2.18	2.16			3.13	3.13	2.74	2.54	
SMC1814LA_152				2.09						2.37	2.18		

## Data Availability

The data presented in this study are available in this article and Supplementary Material.

## Supplementary Materials

The following supporting information can be downloaded at: https://www.mdpi.com/article/10.3390/plants12051041/s1, Table S1: Sucrose and fiber contents in two years (2006 and 2007) and structure analysis in K = 3, among 237 clones of LCP 85-384 population. Table S2: The profile of 135 markers including SSR, AFLP and TRAP with produced polymorphic fragments and coverage/allele. Table S3: List of all associated markers with sucrose and fiber contents by the data in 2006, 2007, and 2006 and 2007 mean in each of the four models, SMR, GLM, MLM and FarmCPU in LCP 85-384 population. Table S4: Genomic prediction (GP) mean accuracy (r-value) and Standard Error for fiber and sucrose using five GP models: Ridge regression best linear unbiased predictor (rrBLUP), Bayes ridge regression (BRR); ’Bayes A’ (BA), ‘Bayes B (BB)’ Bayesian least absolute shrinkage (BL) and selection operator based on All-allele set and Trait-associated allele set.
